# Supplementary material for: Venom Proteomics of Trimeresurus gracilis, a Taiwan-Endemic Pitviper, and Comparison of Its Venom Proteome and VEGF and CRISP Sequences with Those of the Most Related Species
Source: Toxins (Basel). 2023 Jun 22;15(7):408. doi: 10.3390/toxins15070408 (PMC10467061; doi:10.3390/toxins15070408)
Supplement: Supplementary file 1 [file toxins-15-00408-s001.zip › Supplementary_Table_S3_20230616.pdf]

Table S3. Potential bradykinin-potentiating peptides and natriuretic peptides in *Trimeresurus gracilis* venom identified by searching for non-trypsin-digested sequences detected by mass spectrometry.

HPLC fraction	Database accession	Peptide name	Species	Sequence	Protein score	Relative abundance (%)
Fraction 4	gi|547223175	Bradykinin-potentiating peptide/ C-type natriuretic peptide, partial	*Ovophis okinavensis*	VPPDHHAGVGGGGA	42.77	1.49
Fraction 5	gi|547223175	Bradykinin-potentiating peptide/ C-type natriuretic peptide, partial	*Ovophis okinavensis*	VPPDHHAGVGGGGA	56.39	0.96
Fraction 6	gi|1127252755	BATXBPP10	*Bothrops atrox*	PHESPAGGTTAL	31.07	1.08
