# Supplementary figures and images for: Venom Proteomics of Trimeresurus gracilis, a Taiwan-Endemic Pitviper, and Comparison of Its Venom Proteome and VEGF and CRISP Sequences with Those of the Most Related Species
Source: Toxins (Basel). 2023 Jun 22;15(7):408. doi: 10.3390/toxins15070408 (PMC10467061; doi:10.3390/toxins15070408)

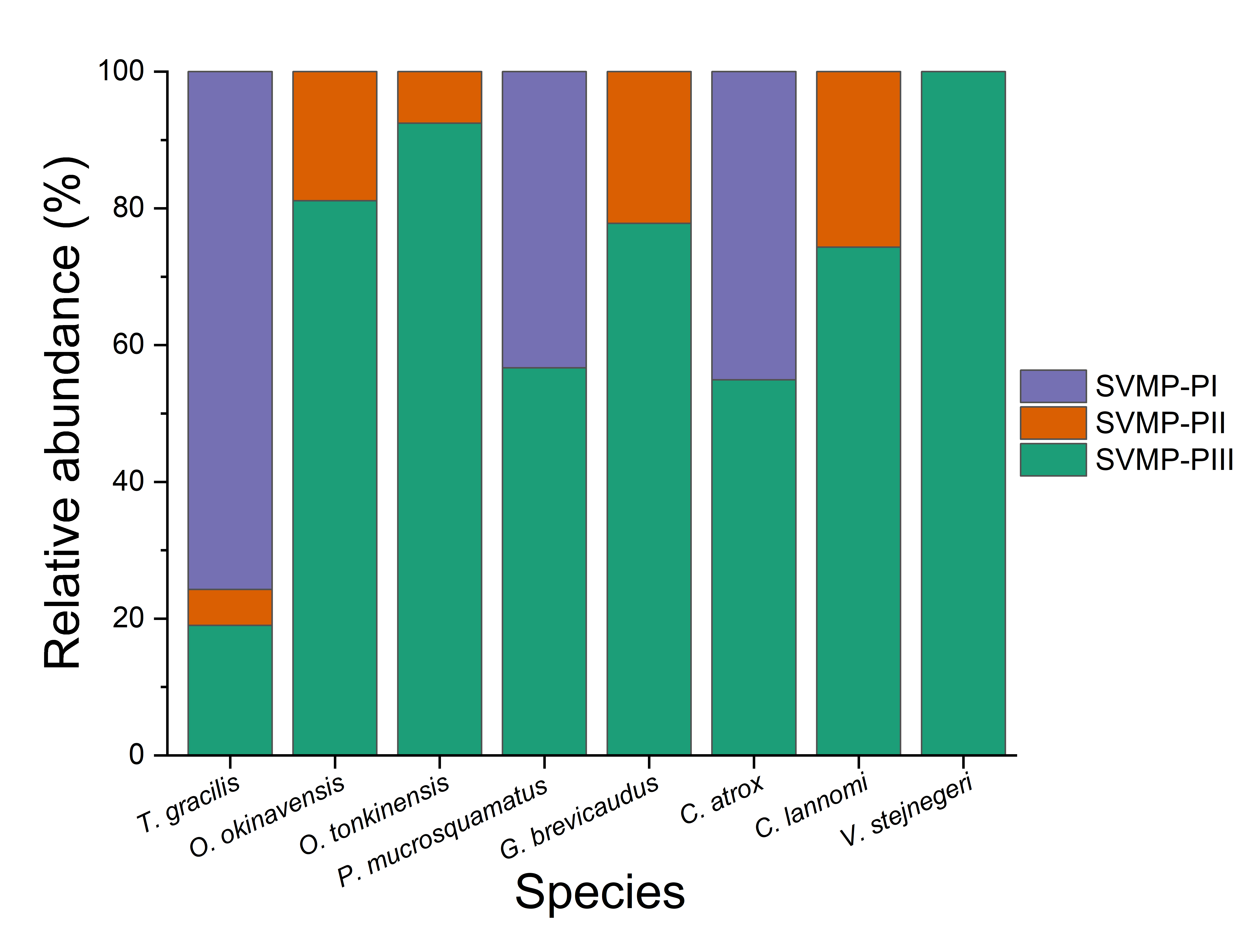

Supplement: Supplementary file 1 [file toxins-15-00408-s001.zip › Supplementary_Fig._S1_20230608.jpg]
